# EMDS-7: Environmental microorganism image dataset seventh version for multiple object detection evaluation

**DOI:** 10.3389/fmicb.2023.1084312

**Published:** 2023-02-20

**Authors:** Hechen Yang, Chen Li, Xin Zhao, Bencheng Cai, Jiawei Zhang, Pingli Ma, Peng Zhao, Ao Chen, Tao Jiang, Hongzan Sun, Yueyang Teng, Shouliang Qi, Xinyu Huang, Marcin Grzegorzek

**Affiliations:** ^1^Microscopic Image and Medical Image Analysis Group, College of Medicine and Biological Information Engineering, Northeastern University, Shenyang, China; ^2^School of Resources and Civil Engineering, Northeastern University, Shenyang, China; ^3^School of Intelligent Medicine, Chengdu University of Traditional Chinese Medicine, Chengdu, China; ^4^International Joint Institute of Robotics and Intelligent Systems, Chengdu University of Information Technology, Chengdu, China; ^5^Shengjing Hospital, China Medical University, Shenyang, China; ^6^Institute of Medical Informatics, University of Lübeck, Lübeck, Germany; ^7^Department of Knowledge Engineering, University of Economics in Katowice, Katowice, Poland

**Keywords:** environmental microorganism, image dataset construction, image analysis, multiple object detection, deep learning

## Abstract

Nowadays, the detection of environmental microorganism indicators is essential for us to assess the degree of pollution, but the traditional detection methods consume a lot of manpower and material resources. Therefore, it is necessary for us to make microbial data sets to be used in artificial intelligence. The Environmental Microorganism Image Dataset Seventh Version (EMDS-7) is a microscopic image data set that is applied in the field of multi-object detection of artificial intelligence. This method reduces the chemicals, manpower and equipment used in the process of detecting microorganisms. EMDS-7 including the original *Environmental Microorganism (EM) images* and the corresponding object labeling files in “.XML” format file. The EMDS-7 data set consists of 41 types of EMs, which has a total of 2,65 images and 13,216 labeled objects. The EMDS-7 database mainly focuses on the object detection. In order to prove the effectiveness of EMDS-7, we select the most commonly used deep learning methods (*Faster-Region Convolutional Neural Network* (Faster-RCNN), YOLOv3, YOLOv4, SSD, and RetinaNet) and evaluation indices for testing and evaluation. EMDS-7 is freely published for non-commercial purpose at: https://figshare.com/articles/dataset/EMDS-7_DataSet/16869571.

## 1. Introduction

### 1.1. Environmental microorganisms

**Figure 1:**
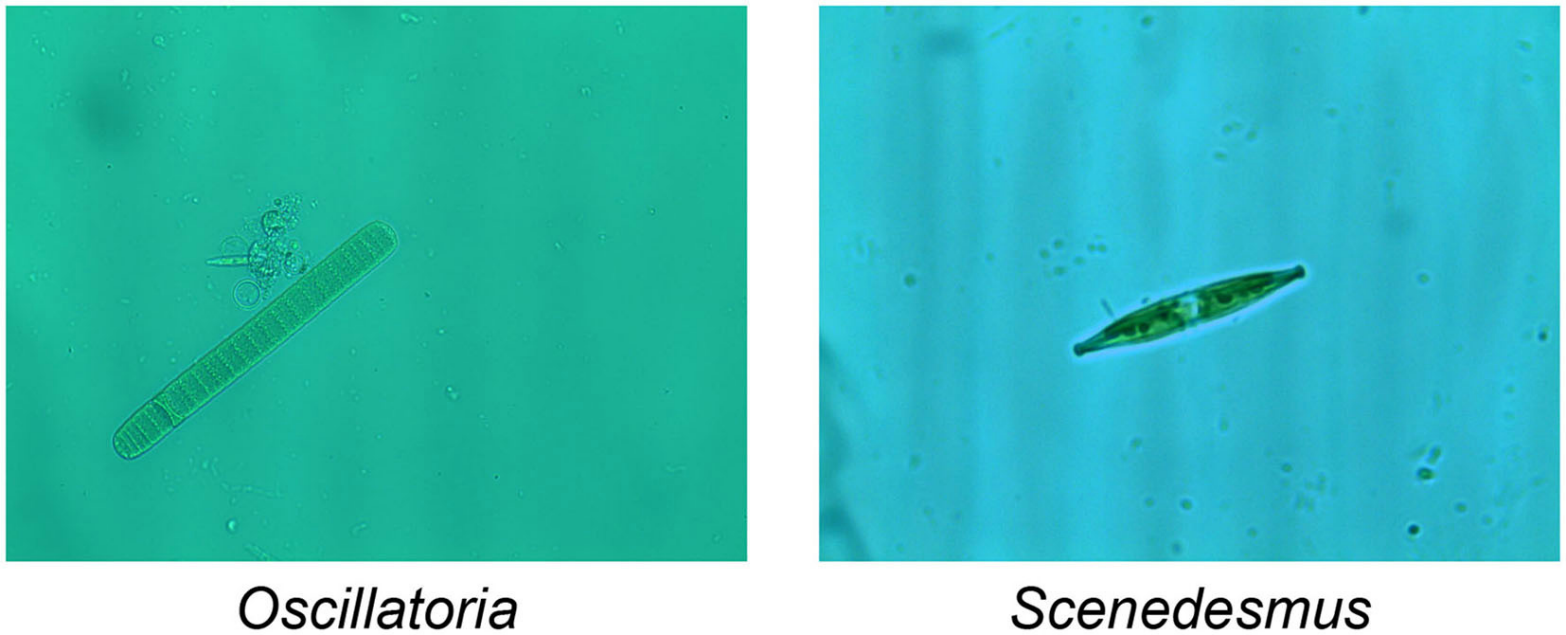
An example of EM images.

Today, Environmental Microorganisms (EMs) are inseparable from our lives (Li et al., 2016). Some EMs are conducive to the development of ecology and promote the progress of human civilization (Zhang et al., 2022b). However, some EMs hinder ecological balance and even cause urban water pollution to affect human health (Anand et al., 2021). For example, *Oscillatoria* is a common EM, which can be observed in various freshwater environments and can thrive in various environments. When it reproduces vigorously, it will produce unpleasant odors, cause water pollution, consume oxygen in the water, and cause fish and shrimp to die of hypoxia (Lu et al., 2021). In addition, *Scenedesmus* is also a freshwater planktonic algae microorganism, which is usually composed of four to eight cells. *Scenedesmus* has strong resistance to organic pollutants, and plays a vital role in water self-purification and sewage purification (Kashyap et al., 2021). The images of the EMs proposed above are shown in Figure 1. But researchers using traditional methods of identifying and analyzing microorganisms will consume a lot of manpower and material resources (Ji et al., 2021). Computer vision analysis method is of great significance, it can help researchers to analyze EMs with higher precision and more comprehensive indicators (Kulwa et al., 2022).

### 1.2. Research background

Automated microscopes and intelligent microscopes are currently a major trend in development, and everyone is working to develop faster, more accurate and objective hardware and software (Abdulhay et al., 2018). Compared with manual identification and observation methods, computer-aided detection methods are more objective, accurate and convenient. With the rapid development of computer vision and deep learning technologies, computer-aided image analysis has been widely used in many research areas, including histopathology image analysis (Chen et al., 2022a,b; Hu et al., 2022a,b; Li et al., 2022), cytopathology image analysis (Rahaman et al., 2020a, 2021; Liu et al., 2022a,b), object detection (Chen A. et al., 2022; Ma et al., 2022; Zou et al., 2022), microorganism classification (Yang et al., 2022; Zhang et al., 2022a; Zhao et al., 2022a), microorganism segmentation (Zhang et al., 2020, 2021a; Kulwa et al., 2023), and microorganism counting (Zhang et al., 2021b, 2022c,d). In addition, with the advancement of computer hardware and the rapid development of computer-aided detection methods. In addition, with the advancement of computer hardware and the rapid development of computer-aided detection methods, the results obtained by computer-aided detection methods in EM testing have been improving. EMs play a very important role in the whole ecosystem. Because of their small size, invisibility to the naked eye, and unknown nature, studying EMs has always been a challenge for humans (Ma et al., 2022). Generally there are four traditional methods of detecting EMs. The first is physical method, which has a high degree of accuracy, but uses very expensive equipment and a time-consuming analytical process (Yamaguchi et al., 2015). The second method is the chemical method, which has a high identification capacity, but is often affected by environmental contamination from chemical agents. The third molecular biological method, by detecting the genes of microorganisms and analyzing them. The accuracy of this method is very high, but at the same time it consumes a lot of consumes a lot of human and material resources (Kosov et al., 2018). The fourth is morphological method, which requires a skilled researcher to observe the shape of EM under a microscope, which is inefficient and time consuming (Li et al., 2019). These four traditional methods of analyzing EM have their own advantages and disadvantages. As deep learning has been widely used in machine vision analysis in recent years (Shen et al., 2017), it can compensate the shortcomings of the four traditional methods while retaining accuracy (Kulwa et al., 2023). However, there are few public EM databases available to researchers today. This hinders the analysis of EM. In recent years with the advent of the EMDS series of datasets, research on artificial intelligence on EM has been carried out. With the update of EDMS-1 to EMDS-6, environmental microbial images are widely used for classification, segmentation, and retrieval (Zhao et al., 2022b). However, there is still a gap for multi-objective EM detection, and EMDS-7 fills this gap as a multi-objective EM dataset. the EMDS details are listed in Table 3.

### 1.3. EM image processing and analysis

Image analysis is the combination of mathematical models and image processing techniques to analyze and extract certain intelligence information (Song et al., 2022). Image processing refers to the use of computers to analyze images. Common image processing includes image denoising, image segmentation, and feature extraction (Liu et al., 2022d). Image noise appears in the process of acquiring and transmitting EMs images (Gonzalez and Woods, 2002). Image denoising can reduce the noise of the EM image while preserving the details of the image (Rahaman et al., 2020b). Besides, in the process of deep learning based EMs image analyzation, we can extract the features of EM images, then send them to the deep learning network model for training, and match them with known data to classify, retrieve and detect EMs (Liu et al., 2022c). In addition, EM images can also be applied in the field of image segmentation to separate microorganisms from the complex background of the image (Pal and Pal, 1993). Meanwhile, EM images can be used in the field of EM object detection. First, we can frame and mark the known EM objects in the original image, and then transfer the image to the object detection model for feature extraction and network training (Zhang et al., 2021b). Finally, the trained model can be applied for object detection of EMs.

### 1.4. Contribution

EMs are one of the important indicators for investigating the environment, so it is very essential to collect EM data and information (Kosov et al., 2018). Environmental Microorganism Image Dataset SeventhVersion (EMDS-7) are all taken from urban areas, which can be used to monitor the pollution of the urban water environment. Furthermore, due to the constant changes in conditions such as temperature and humidity, EMs are very sensitive to these conditions, so the biomass of EMs are easily affected (Rodriguez et al., 2020). It is difficult to collect enough EM images. Currently, there are some EM data sets, but many of them are not open source. EMDS-7 is provided to researchers as an open source data set. In addition, we prepare high-quality corresponding object label files of EMDS-7 for algorithms and model evaluation. The label file of EMs can be directly used in multiple object detection and analysis. EMDS-7 has a variety of EM images, which provides sufficient data support for EMs object detection and achieves satisfactory detection results. Researchers can apply many artificial intelligence methods instead of traditional analysis methods to analyze microorganisms in EMDS-7.

The main contributions of this paper are as follows.

(1) EMDS-7 is available to researchers as an open source dataset that helps to analyze microbial images.
(2) High quality corresponding object label files of EMDS-7 for algorithm and model evaluation. Label files of EMs can be directly used for detection and analysis of multiple objects.
(3) Performance analysis of multiple object detection models on EMDS-7 is provided, which facilitates further ensemble learning.

## 2. Dataset information of EMDS-7

**Figure 2:**
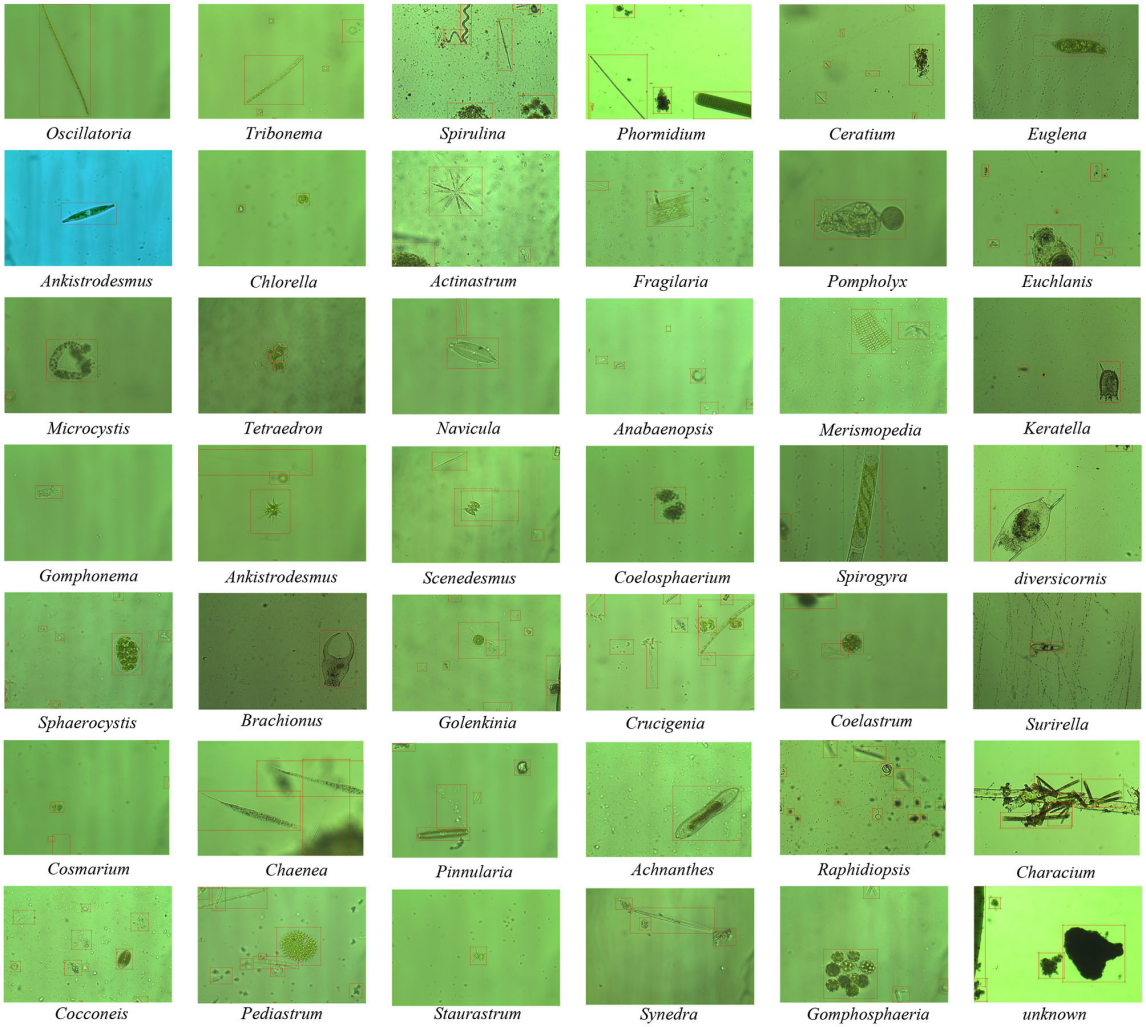
An example of EM images in EMDS-7 (The red boxes are labeled EM objects).

EMDS-7 consists of 2365 images of 42 EM categories and 13216 labeled objects. The EM sampling sources are images taken from different lakes and rivers in Shenyang (Northeast China) by two environmental biologists (Northeastern University, China) under a 400 × optical microscope from 2018 to 2019. Then, four bioinformatics scientists (Northeastern University, China) manually prepared the object labeling files in “.XML” format corresponding to the original 2,365 images from 2020 to 2021. In the EM object labeling files, 42 types of EMs are labeled by their categories. In addition, the unknown EMs and impurities are marked as Unknown, and a total of 13,216 labeled objects are obtained. In Table 1 we list the 42 categories of EMs included in EMDS-7. Also for a more visual presentation of our dataset, we list in the table detailed information about each microorganism category, such as the number of original images of each microorganism category, the total number of annotations of each microorganism category and the visible characteristics of each microorganism category. Figure 2 shows examples of 41 types of EMs and unknown objects in EMDS-7. The labeled files of EMDS-7 images are manually labeled base on the following two rules:

**Table 1:** Basic information of 42 EM classes in EMDS-7.

**Classes**	**NoOI**	**NEMo**	**VC**	**Classes**	**NoOI**	**NEMo**	**VC**
*Oscillatoria*	41	178	Cylindrical	*Staurastrum*	9	9	Multi-radial symmetry
*Ankistrodesmus*	5	45	Cell needle	*Phormidium*	276	1,216	Plant body gelatinous or leathery
*Microcystis*	307	826	Spherical masses	*Fragilaria*	55	59	Lanceolate
*Gomphonema*	87	108	Linear-lanceolate	*Anabaenopsis*	22	37	Filaments
*Sphaerocystis*	55	53	Cell sphere	*Coelosphaerium*	77	165	Group glue is generous and transparent
*Cosmarium*	17	28	cell side flattened	*Crucigenia*	9	16	Micelles with many cells
*Cocconeis*	14	15	Flat, oval cells	*Achnanthes*	18	19	Shell surface linear lanceolate
*Tribonema*	49	88	Yellow-green cotton-like	*Synedra*	77	206	Shell needle
*Chlorella*	80	155	Small round or slightly oval	*Ceratium*	23	24	Flat back and abdomen
*Tetraedron*	25	66	Flat or pyramidal	*Pompholyx*	49	51	Carapace Oval or Shield Shape
*Ankistrodesmus*	64	84	Needle to spindle	*Merismopedia*	33	38	Flat group
*Brachionus*	113	144	quilt is wider and square	*Spirogyra*	89	134	Strip, spiral
*Chaenea*	6	13	Cylindrical or spindle	*Coelastrum*	29	30	Spherical, oval or truncated pyramid
*Pediastrum*	95	105	Disc or star	*Raphidiopsis*	9	19	Curved
*Spirulina*	18	73	Spiral	*Gomphosphaeria*	58	79	Oval tiny groups
*Actinastrum*	23	181	Wide round or pointed	*Euglena*	81	81	Spindle to needle
*Navicula*	75	90	Shell surface fusiform or oval	*Euchlanis*	14	13	Oval or pear-shaped
*Scenedesmus*	86	139	Oval or spindle	*Keratella*	65	69	Irregular spines
*Golenkinia*	60	279	Irregularly slender bristles	*diversicornis*	89	99	Feet have toes and quilt
*Pinnularia*	36	38	Oval to boat	*Surirella*	22	37	False shell seam
*unknown*		8088	Unknown EM and impurities	*Characium*	5	19	Spindle

NoOI, Number of original images; NEMo, Number of EM object; VC, Visible characteristics.

Rule-A: All identifiable EMs that appear completely or more than 60% of their own in all images are marked with category labels corresponding to 41 categories.

Rule-B: Unknown EMs that are < 40% of their own in all images and EMs other than 41 categories in this database. In addition, some obvious impurities in the background of the image are marked as unknown.

EMDS-7 is freely published for non-commercial purpose at: https://figshare.com/articles/dataset/EMDS-7_DataSet/16869571.

## 3. Object detection methods for EMDS-7 evaluation in this paper

In this paper, five object detection models are selected to demonstrate the effectiveness of EMDS-7. The five models include one-stage detection model and two-stage detection models. Among them, the one-stage object detection algorithms, which are characterized by one-step, only need to feed the network once to predict all the bounding boxes, this type of algorithms are relatively low accuracy, but relatively fast, and very suitable for mobile. We choose YOLOv3, YOLOv4, SSD, and RetinaNet as one-stage object detection models in this paper. In contrast, two-stage model will first generate region proposals (regions that may contain objects) and then classify each region proposals. This type of algorithms is relatively accurate, but relatively slow because they require multiple runs of the detection and classification process. We choose *Faster-Region Convolutional Neural Network* (Faster-RCNN) as a two-stage object detection model in this paper. Finally, we analyze the variability of different deep learning networks in EMDS-7 from the above two directions.

### 3.1. YOLOv3

The object detection model of the YOLO series is a one-stage detection network, which can locate and classify the objects at the same time. The advantage is that the training speed is fast with less time-consuming. One of the most types is YOLOv3. Joseph Redmon and others used the new basic network darknet-53 in the backbone of YOLOv3 for physical sign extraction (Redmon et al., 2016). It contains 53 convolutional layers and introduces a residual structure so that the network can reach a deep level while avoiding the problem of gradient disappearance. In addition, darknet-53 removes the pooling layer and uses a convolutional layer with a step size of 2 to reduce the dimensionality of the feature map, which can maintain the information transmission better. And YOLOv3 also has excellent structures such as anchor and FPN (Lin et al., 2017a).

### 3.2. YOLOv4

YOLOv4 is an improved version based on YOLOv3, which adds CSP and PAN structures (Bochkovskiy et al., 2020). The backbone network of YOLOv3 is modified to CSPDarkne53, and add an spp (spatial pyramid pooling) idea behind the backbone network to expand the receptive field, using 1 × 1, 5 × 5, 9 × 9, 13 × 13 as the largest Pooling, multi-scale fusion, and improve the accuracy of the model. At the same time, in the neck network of YOLOv4, there are Feature Pyramid Network (FPN) (Lin et al., 2017a), Path Aggregation Network (PAN), BiFPN, and NAS-FPN, which can collect different feature maps more effectively.

### 3.3. SSD

SSD is another striking object detection network after YOLO. SSD has two major advantages (Liu et al., 2016). First, SSD extracts feature maps of different scales for detection. Large-scale feature maps can be used to detect small objects, while small-scale feature maps can be used to detect large objects. Second, SSD uses different Prior boxes (Prior boxes, Default boxes, Anchors) for scale and aspect ratio. It follows the method of direct regression box and classification probability in YOLO, and uses anchors to improve recognition accuracy referring to Faster R-CNN. By combining these two networks, SSD balances the advantages and disadvantages of Faster R-CNN and YOLO.

### 3.4. RetinaNet

The RetinaNet object detection model is also a one-stage object detection network. RetinaNet essentially consists of a backbone network (BackBone) and two subnets (SubNet) (Lin et al., 2017b). The backbone network is responsible for calculating the convolution feature map on the entire input image, which is composed of the ResNet residual network and the FPN feature pyramid network. The two sub-networks use the features extracted from the backbone network to achieve their respective functions (Lin et al., 2017a). The first sub-network completes the classification task; the second sub-network completes the bounding box regression task.

### 3.5. Faster RCNN

Faster RCNN generates candidate frames based on the Anchor mechanism by adding a region proposal networks (RPN), and finally integrates feature extraction, candidate frame selection, frame regression, and classification into one network, thereby the detection accuracy and efficiency can be effectively improved (Ren et al., 2015). Faster RCNN performs classification and detection of foreground and background in the RPN network structure, optimizes the complexity of picking samples, makes positive and negative samples become more balanced, and then focuses on some parameters for classification training. For the first stage of object detection, it has to do both localization and classification, and there is no clear division of labor which part is dedicated to classification and which part is dedicated to regression of prediction frames, so that the learning difficulty increases for each parameter. Therefore, the classification difficulty of the second stage training will be much easier than the first stage target detection to do mixed classification and prediction frame regression directly.

## 4. Evaluation of deep learning object detection methods

**Figure 3:**
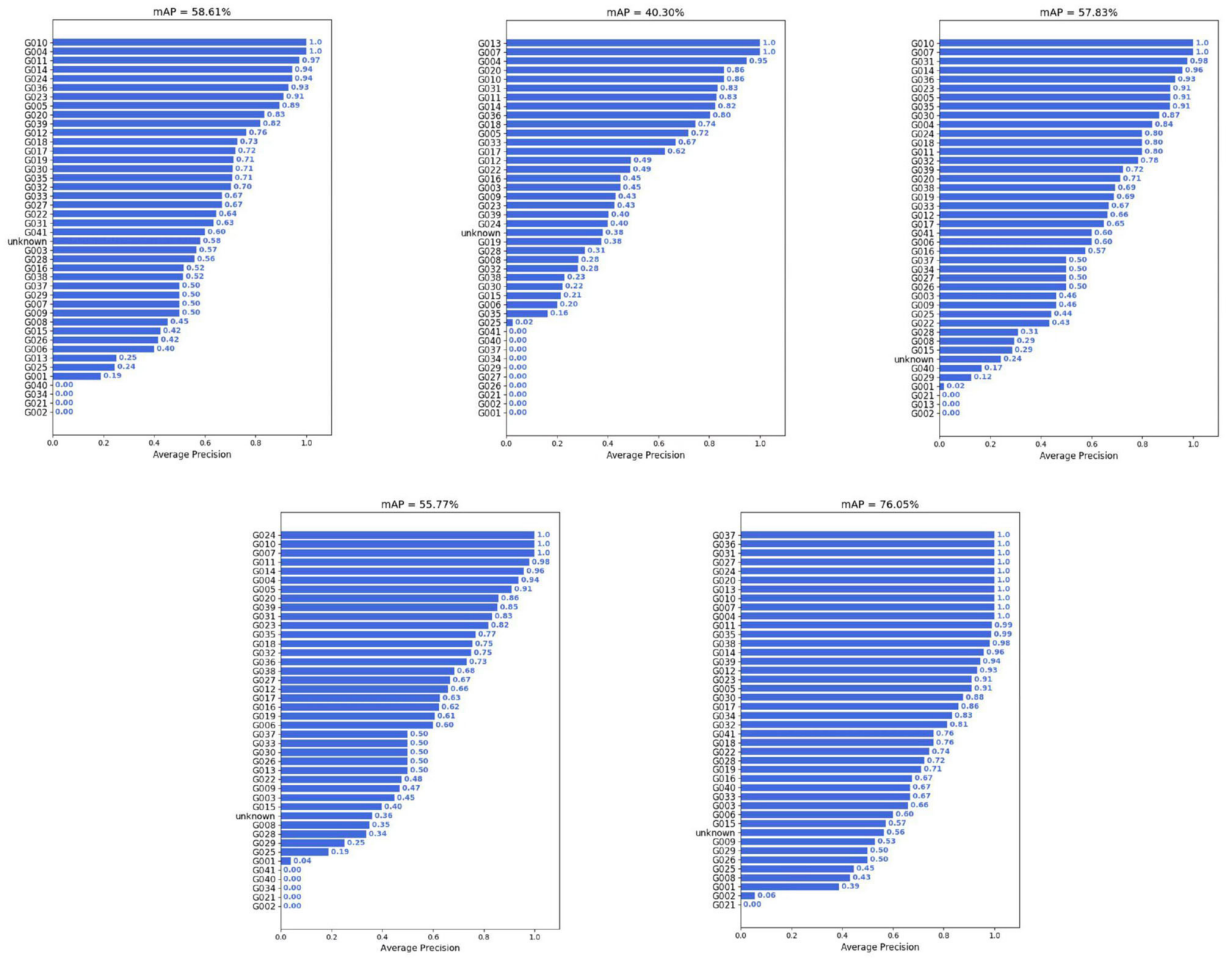
Each category of EM object detection prediction AP value in EMDS-7.

**Figure 4:**
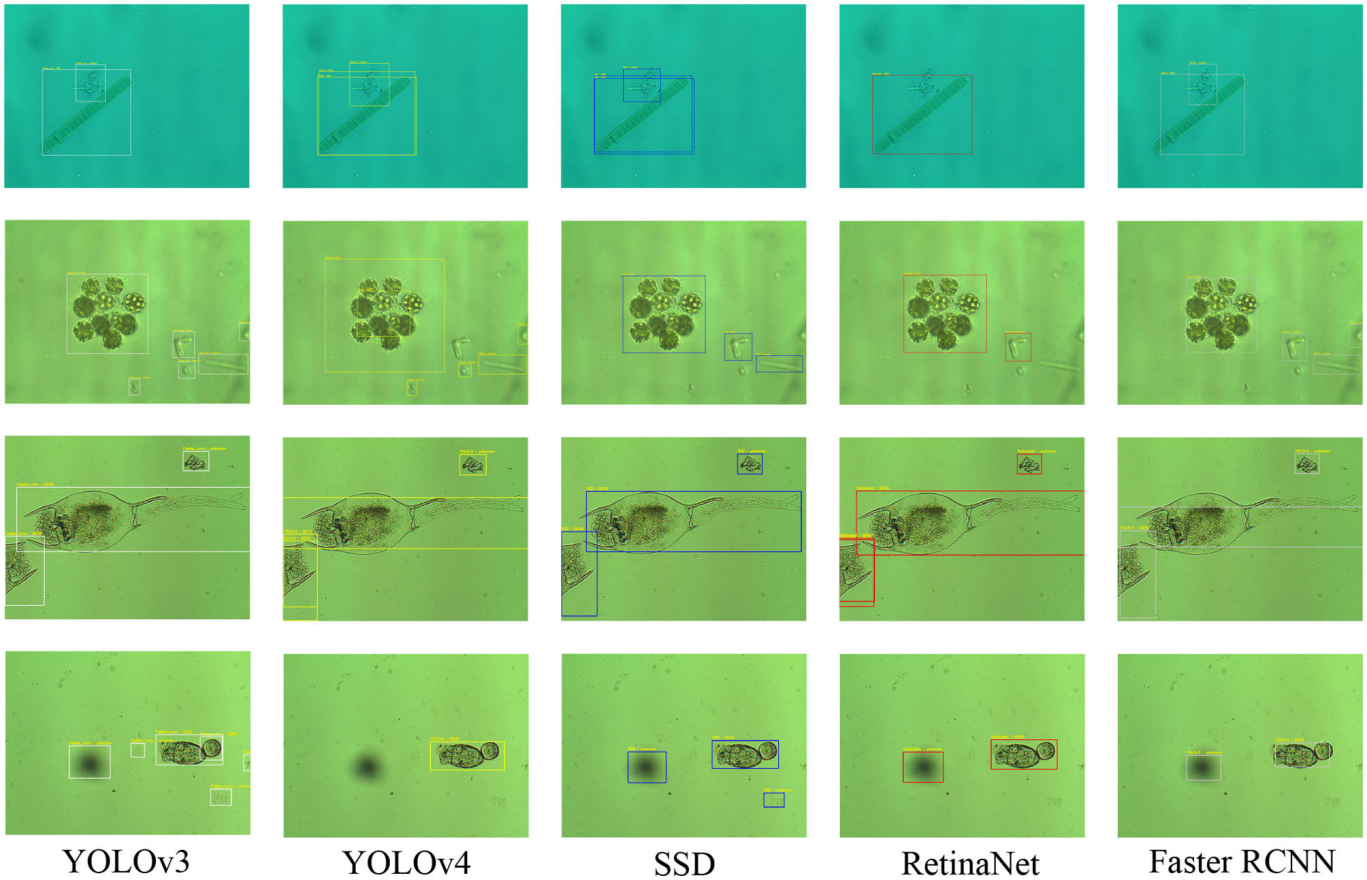
Five object detection model prediction results in EMDS-7 (the microorganisms predicted by the five models are marked with five color boxes respectively. YOLOv3, white; YOLOv4, yellow; SSD, blue; RetinaNet, red; Fast RCNN, gray).

Object detection is an important part of image analysis (Sun et al., 2020). To prove the effectiveness of EMDS-7 in object detection and evaluation, we use five different deep learning object detection models to detect EMs in the EMDS-7 data set. The five models are YOLOv3 (Redmon et al., 2016), YOLOv4 (Bochkovskiy et al., 2020), SSD (Liu et al., 2016), RetinaNet (Lin et al., 2017b), and Faster RCNN (Ren et al., 2015). Because the number of images of each category of EM in the EMDS-7 data set is different, we divide each category of Ems data set into the training, validation and test set according to 6:2:2 to ensure each sets has 42 types of EMs (Chen et al., 2021). We train EMDS-7 for five different kinds of deep learning object detection model, and then, respectively, predict the images of the test set (Wang et al., 2022). We calculate the number of EM objects in 456 EMS images. We set the threshold of the predictive frame confidence to be 0.5, when the model predicts the object's confidence is >0.5, the prediction box is displayed, and Intersection over Union (IOU) is set to 0.3. In Figure 3, we summarize the Average Precision (AP) value of each class of EMs. Analysis of predict results is shown in Table 2. We also illustrate the location of the prediction box in the EMS image, and some samples are shown in Figure 4.

**Table 2:** A comparison of the object detection results on test set of EMs.

		**YOLOv3**		**YOLOv4**		**SSD**		**RetinaNet**		**Faster RCNN**	
**EMS**	**GTO**	**tp**	**fp**	**tp**	**fp**	**tp**	**fp**	**tp**	**fp**	**tp**	**fp**
*Oscillatoria*	26	6	9	0	0	1	1	1	0	14	25
*Ankistrodesmus*	9	0	1	0	0	0	1	0	0	1	2
*Microcystis*	151	105	95	87	37	83	35	94	31	118	112
*Gomphonema*	19	19	6	18	1	16	2	18	2	19	5
*Sphaerocystis*	11	10	3	8	1	10	0	10	0	10	0
*Cosmarium*	5	2	0	1	0	3	0	3	1	3	2
*Cocconeis*	2	1	0	2	0	2	0	2	0	2	1
*Tribonema*	17	8	7	5	3	5	0	8	7	8	5
*Chlorella*	34	18	11	15	4	16	3	15	1	20	24
*Tetraedron*	7	7	5	6	0	7	0	7	1	7	3
*Ankistrodesmus*	13	13	5	12	4	11	2	12	1	13	6
*Brachionus*	25	21	13	13	4	17	2	25	9	25	19
*Chaenea*	2	1	1	2	0	0	0	1	0	2	1
*Pediastrum*	24	23	3	20	1	23	0	20	2	23	1
*Spirulina*	14	8	7	3	0	4	0	9	2	8	6
*Actinastrum*	40	23	14	18	0	23	0	29	3	29	14
*Navicula*	16	12	5	10	3	12	6	15	6	14	7
*Scenedesmus*	25	19	10	19	3	20	1	18	0	19	7
*Golenkinia*	41	32	35	20	8	29	8	29	9	31	25
*Pinnularia*	7	7	4	6	0	5	0	6	1	7	3
*Staurastrum*	3	0	0	1	1	0	0	0	0	0	1
*Phormidium*	234	188	151	147	73	4	1	142	32	194	127
*Fragilaria*	11	10	2	6	3	21	20	10	3	10	3
*Anabaenopsis*	5	5	2	2	0	2	0	9	2	5	1
*Coelosphaerium*	42	11	2	1	0	2	2	6	3	21	12
*Crucigenia*	4	2	1	0	0	11	3	2	0	2	1
*Achnanthes*	3	2	1	0	0	2	2	3	0	3	0
*Synedra*	34	23	29	16	14	11	3	15	1	27	21
*Ceratium*	4	2	2	0	0	1	1	1	1	2	0
*Pompholyx*	9	7	5	2	0	8	4	8	3	8	5
*Merismopedia*	6	4	1	5	0	6	1	5	0	6	1
*Spirogyra*	25	19	16	12	16	20	4	21	2	21	9
*Coelastrum*	6	4	1	4	0	4	1	3	0	4	0
*Raphidiopsis*	2	0	0	0	0	1	1	0	0	2	1
*Gomphosphaeria*	14	10	2	5	8	13	2	11	3	14	6
*Euglena*	16	15	6	14	4	15	1	12	1	16	1
*Euchlanis*	2	1	0	0	0	1	0	0	0	2	0
*Keratella*	13	7	4	3	1	9	1	9	1	13	4
*diversicornis*	18	17	17	13	14	14	3	19	11	17	4
*Surirella*	6	0	0	0	0	1	0	1	1	4	3
*Characium*	5	3	0	0	0	3	1	0	0	4	2
*unknown*	1429	1068	1126	610	177	371	66	622	146	1049	1585

GTO, ground ture object; tp, ture positive, fp, flase positive (In [%]).

In Figure 3 and Table 2 we can see that the EMDS-7 data set performs well in the task of object detection, and most of the EMs can be accurately identified. Meanwhile, different object detection models differ in the object detection effect of EMDS-7, which proves that EMDS-7 dataset provide performance analysis of different networks. In Figure 3 we calculate five models of the Mean Average Precision (MAP), which the Faster RCNN value of 76.05%, the YOLOv3 value of 58.61%, the YOLOv4 value of 40.30%, the SSD value of 57.83%, the RetinaNet value of 57.96%. We can see that Faster RCNN has the highest detection performance of EMDS-7. YOLOv3, SSD, RetinaNet model predicts that the MAP value is similar. The lowest is YOLOv4. Our EMDS-7 dataset is prepared for tiny object detection tasks, which is different for regular images. Although YOLOv4 is an improved version of YOLOv3, their structures have different performance for multi-scale small object inspection. YOLOv3 is also widely used in industry, and because v4 adds CSP and PAN structures to YOLOv3, YOLOv3 is less than YOLOv4 in terms of computational resources. However, some EMS category predictive AP values can reach 100%, while some kind of AP values are 0%. We find that there are little AP value is 100% or 0%, and the category of EMs in the image is basically consistent and the EMs characteristics are relatively large. In addition, different models are different from the method of extracting features, so differentiation occurs when model training. For example, in the object detection of Euglena category, the predictive sample has only two, and the AP value is 100% in the FASTER RCNN, but in the RetinaNet is 0%. We list the prediction results of the five models in Table 2. For example, the true sample size of the *Microcystis* prediction set was 151, of which the faster-RCNN model correctly identified 118, while the incorrect identification was 112. The ability of Faster RCNN and YOLOv3 to correctly detect *Microcystis* is higher than the other three models. However, the number of *Microcystis* incorrectly detecte by YOLOv4, SSD, and RetinaNet is smaller than that of Faster RCNN and YOLOv3. The Faster RCNN and YOLOv3 models we trained are more capable of learning to detect *Microcystis*, so that the fp and tp are both higher. Also among the five models, the Faster-RCNN had the highest number of correct identifications for *Microcystis* species, and the SSD had the lowest number of correct identifications at 83. Table 2 describes the predictions of the five models for each category of EMs. In synthesis, the EMDS-7 database can provide analytical performance for different object detection models.

## 5. Discussion

Table 3 shows the development history process of the EMDS versions. Seven versions of the EMDS were published, and different versions of the dataset have different features. Both EMDS-1 and EMDS-2 contain 10 classes of EMs with 20 original images and 20 GT images per class, which can be used for image classification and segmentation. No new features were added to EMDS-3. However, we have extended five classes of EMs. Compared with EMDS-3, EMDS-4 has been extended with six new classes of EMs and added a new image retrieval function. In EMDS-5, 420 single-object GT images and 420 multi-object GT images are prepared, respectively. Thus, EMDS-5 supports more functions. Based on EMDS-5, EMDS-6 adds 420 original images and 420 multi-object GT images. With the support of more data volume, EMDS-6 can realize more functions in a better and more stable way. EMDS-7 is specially applied to the object detection dataset with more sufficient data volume than the previous versions, so that the EM object function can be realized in a better and more stable way. In addition, we have prepared label files corresponding to each image.

**Table 3:** EMDS history versions and latest versions.

**Dataset**	**ECN**	**OIN**	**GTIN**	**Functions**	**Dataset link**
EMDS-1 (Li et al., 2013)	10	200	200	IC, IS	
EMDS-2 (Li et al., 2013)	10	200	200	IC, IS	
EMDS-3 (Li et al., 2016)	15	300	200	IC, IS	
EMDS-4 (Kosov et al., 2018)	21	420	420	IC, IS, IR	https://research.project-10.de/em-classiffication/
EMDS-5 (Li et al., 2021)	21	420	840 (S,M 420)	ID, IED, SoIS, MoIS, SoFE, MoFE, IR	https://github.com/NEUZihan/EMDS-5
EMDS-6 (Zhao et al., 2022b)	21	840	840	ID, IC, IS, IFE, IOD	https://figshare.com/articles/dataset/EMDS6/17125025/1
EMDS-7 [In this article]	42	2365	2365 (‘xml')	IOD	https://figshare.com/articles/dataset/EMDS-7_DataSet/16869571

IC, Image Classification; IS, Image Segmentation; SoIS, Single-object Image Segmentation; MoIS, Multi-object Image Segmentation; SoFE, Single-object Feature Extraction; MoFE, Multi-object Feature Extraction; IR, Image Retrieval; IFE, Image Feature Extraction; IOD, Image Object Detection; IED, Image Edge Detection; ID, Image denoising; ECN, EM Class Number; OIN, Original Image Number; GTIN, Ground Truth Image Number; S, Single Object; M, Multiple object.

## 6. Conclusion and future work

EMDS-7 is an object detection data set containing 42 types of EMs, which contains the original image of EMs and object label data for corresponding EMs. EMDS-7 labeled 15342 EMs. At the same time, we further add some deep learning object detection experiments to the EMDS-7 database to prove the effectiveness. During the object detection process, we divide the data set according to 6: 2: 2 for train, validation and test sets. We use five different deep learning object detection methods to test EMDS-7 and use multiple evaluation indices to evaluate the prediction results. According to our experiments, EMDS-7 behaves differently in different deep learning models, so EMDS-7 can provide an analysis of the performance of different networks. Meanwhile, in this paper EMDS-7 has the highest accuracy on the Faster RCNN prediction test set, and its map is 76.05%, which has achieved good performance in the deep learning of object detection.

In the future, we will enlarge the category of EMs to increase the number of images of each EM. Make each class data balanced and sufficient. We hope to use the EMDS-7 database to achieve more features in the future.

## Data availability statement

The datasets presented in this study can be found in online repositories. The names of the repository/repositories and accession number(s) can be found below: https://figshare.com/articles/dataset/EMDS-7_DataSet/16869571.

## Author contributions

HY: data, experiment, and writing. CL: corresponding author, team leader, method, data, experiment, and writing. XZ: corresponding author, data, and writing. BC, JZ, and PM: data. PZ: experiment. AC and HS: method. TJ, YT, and SQ: result analysis. XH and MG: proofreading. All authors contributed to the article and approved the submitted version.
